# Anti-Mullerian Hormone (AMH) and adenomyosis: Mini-review of literature of the last 5 years

**DOI:** 10.3389/fendo.2022.1014519

**Published:** 2022-08-31

**Authors:** Ferdinando Antonio Gulino, Valentina Dilisi, Stella Capriglione, Francesco Cannone, Francesco Catania, Francesco Giuseppe Martire, Attilio Tuscano, Marianna Gulisano, Valentina D’Urso, Alessandra Di Stefano, Monia Caterina Cimino, Maurizio Filippini, Silvia Latella, Margaret Sammarini, Giulia Musmeci, Marco Antonio Palumbo

**Affiliations:** ^1^ Department of Obstetrics and Gynecology, Azienda di Rilievo Nazionale e Alta Specializzazione (ARNAS) Garibaldi, Catania, Italy; ^2^ Department of Medical Surgical Specialties, Gynecology and Obstetrics Section, University of Catania, Catania, Italy; ^3^ Department of Obstetrics and Gynecology, Ospedale “Santa Maria Alla Gruccia”, Montevarchi, Italy; ^4^ Gynecological Unit, Department of Surgical Sciences, University of Rome “Tor Vergata”, Roma, Italy; ^5^ Department of Obstetrics and Gynaecology, Ospedale di Stato, Cailungo, San Marino; ^6^ Department of Hospital Pharmacy, San’Elia Hospital, Caltanissetta, Italy

**Keywords:** adenomyosis, amh, fertility, adenomyomectomy, hifu (high intensity focus ultrasound)

## Abstract

**Introduction:**

Adenomyosis is a form of endometriosis characterized by the presence of endometrial tissue in the myometrium. The correlation between anti-Mullerian hormone (AMH) expression and adenomyosis is unclear. Few studies investigated this possible correlation with promising results. The aim of this mini-review is to illustrate the potential prognostic and therapeutic role of AMH in adenomyosis.

**Materials and methods:**

A study protocol was completed conforming to the Preferred Reporting Items for Reviews and Meta-Analyses (PRISMA) guidelines for systematic reviews. We performed an electronic databases search from each database’s inception from August 2017 to August 2022 for full-text articles and published abstracts. For database searches, the following main keywords were the following text words: “adenomyosis” or “uterine endometriosis” [Mesh] AND “AMH” or “anti-mullerian hormone”.

**Results:**

From the literature search, 8 abstracts of studies were retrieved and independently screened for inclusion by three authors. It was found that the most common therapeutic strategies (such as adenomyomectomy and high-intensity focused ultrasound (HIFU) do not alter AMH levels. Moreover, a higher expression of the AMH receptor II was observed in adenomyotic tissue, hence a possible therapeutic use of AMH was hypothesized.

**Conclusion:**

The available evidence shows an unclear relationship between adenomyosis and AMH. Probably, women with adenomyosis have lower levels of AMH and the surgical treatment (adenomyomectomy, HIFU) does not alter this characteristic, therefore in all of them, ovarian function is not influenced.

## Introduction

Adenomyosis is a form of endometriosis characterized by the presence of endometrial tissue in the myometrium. It is not simple to estimate the real impact of this disease, but recent studies suggest a prevalence of around 20-35% (1). Clinically, it can be asymptomatic or it can cause menometrorrhagia, dysmenorrhea, pelvic pain, dyspareunia and subfertility (1). Until now, it was possible to make a diagnosis of adenomyosis only by histological examination after hysterectomy; but nowadays a careful study by Trans-Vaginal Ultrasound (TVS) and Magnetic Resonance Imaging (MRI) could allow diagnosing this condition without the need for surgery (2). The relationship between adenomyosis and fertility is controversial, but the last evidence shows a decreased fertility in affected women (3). One of the most studied parameters of fertility is the anti-Mullerian hormone (AMH). AMH is a glycoprotein hormone and is a negative regulator of women’s folliculogenesis. If this pathway is altered, two causes could lead to infertility: primary ovarian insufficiency (POI) and polycystic ovary syndrome (PCOS) (4).

The aim of this mini-review is to illustrate the potential prognostic and therapeutic role of AMH in adenomyosis, considering the scientific works published in the last 5 years.

## Materials and methods

A study protocol was completed conforming to the Preferred Reporting Items for Reviews and Meta-Analyses (PRISMA) guidelines for systematic reviews. Literature Search: studies were identified through a systematic literature search on online databases: PubMed, Medline, Embase, Cochrane Library and Web of Science. We performed an electronic databases search from each database’s inception since August 2017 to August 2022 for full-text articles and published abstracts. We did not limit the search by language, geographic origin, date of publication, or study type. Studies were limited to humans and animals’ studies were excluded. For database searches, the following main keywords were the following text words: “adenomyosis” or “uterine endometriosis” [Mesh] AND “AMH” or “anti-mullerian hormone”.

## Results

From the literature search, 8 abstracts of studies were retrieved and independently screened for inclusion by three authors. The information extracted included study general information (title, author, year and journal), study characteristics (type of study design, outcome), and interventions. We differentiated the results related to adenomyosis treatment and the results of the studies related to AMH receptor in adenomyosis.

### AMH and adenomyosis treatment

Adenomyosis could be treated by medical or surgical treatment. Medical treatment includes oral contraceptives, progesterone-releasing intrauterine devices, ulipristal acetate, non-steroidal anti-inflammatory drugs and gonadotropin-releasing hormone agonists (5). These methods relieve symptoms, but the definitive treatment is surgical, first of all, hysterectomy. However, this option cannot be considered for patients who desire a pregnancy; in this case, other viable surgical treatments are adenomyomectomy and HIFU. Uterine-sparing surgery for adenomyosis is a complex procedure because it is associated with heavy intraoperative bleeding and led to a high risk of spontaneous uterine rupture in the subsequent pregnancy. Furthermore, it is difficult to distinguish healthy myometrium from adenomyotic tissue (6).

Another alternative technique to treat adenomyosis in young women is USgHIFU (ultrasound-guided high-intensity focused ultrasound). This procedure has less complications and shorter hospitalization than adenomyomectomy; but, like surgery, the symptoms could come back considering the unclear boundaries of the adenomyotic lesion (5). Whether surgical removal of adenomyosis can affect fertility outcomes or not is still under debate.

The aim of this review is to compare the AMH levels in serum before and after adenomyosis treatment. Pedachenko and colleagues (7) analyzed 149 patients in a cross-sectional study and divided them into 2 groups: group A composed of 72 infertile women with endometriosis (adenomyosis, retrocervical adenomyosis and ovarian endometriomas) and group B composed by 77 infertile women without endometriosis. It was observed that the AMH level, without surgical treatment, was lower in group A (women with endometriosis) than in group B, particularly in the subgroup with ovarian endometriomas.

Two studies evaluated how AMH levels change after adenomyomectomy. Limei Ji et al. (8) compared two techniques to treat adenomyosis and assessed the associated change of AMH after first menstruation, after 12 months and after 24 months. The first technique is laparoscopic adenomyomectomy with the double/multiple-flap (n= 76) and the second one is double/multiple-flap adenomyomectomy combined with temporary occlusion of the bilateral uterine artery and utero-ovarian vessels (n=79). Levels of AMH did not differ significantly between the two groups and throughout the follow-up period. In addition, Won et al. (6) analysed, in a retrospective study, 43 women that underwent adenomyomectomy and desired a pregnancy. 15 patients, after surgery, had a pregnancy. Younger age and higher AMH levels were found to be significant predictive factors for a successful pregnancy.

The other two studies instead evaluated AMH levels after HIFU. AMH serum levels were evaluated in 34 women with a diagnosis of adenomyosis before and 6 months after HIFU and no significant difference was observed (9). In another study, Keserci et al. observed that, among 66 patients with adenomyosis, AMH levels before and 6 months after treatment were not statistically different (10).

### AMH receptor in adenomyosis

Kim et al. (11) examined 58 uterus specimens obtained after hysterectomy for myomas and/or adenomyosis. Immunohistochemical staining and reverse transcriptase-polymerase chain reaction (RT-PCR) were used to identify AMH receptor II (AMHRII) in each tissue. It was found that AMHRII protein and AMHRII mRNA were much more strongly expressed in adenomyotic tissue (11). This finding could suggest that AMH may be evaluated as a biological modulator or even as a possible therapeutic agent on AMHRII expressing-adenomyosis.

## Discussion

Adenomyosis is a pathological condition which could be located in different sites of myometrium, ranging from focal to diffuse adenomyosis. It could be manifest as a nodular lesion called an adenomyoma. The exact prevalence of adenomyosis is difficult to accurately assess due to the need of histological confirmation (12). However, the study of prevalence made by histological diagnosis has a large selection bias considering that women undergoing hysterectomy are in advanced age and with severe clinical symptoms (12). A scoring system which differentiates the grades and the type of adenomyosis and its extension inside the uterus was proposed (13), and it was useful not only for the diagnosis but also for treatment (14).

Some previous studies have demonstrated that serum AMH value was temporarily reduced after myomectomy, but it came back to its presurgical value in a short time. AMH value significantly decreased for 3 months after total surgical hysterectomy. This surgical operation could affect reserve and function of ovaries significantly compared with myomectomy (15). In another scientific work on the effect of uterine artery embolization (UAE) and hysterectomy on ovaries, AMH values were significantly lower during the follow-up period in both treatment groups (UAE and hysterectomy) in comparison to normal AMH levels due to ageing: this study demonstrated that both UAE and hysterectomy could affect ovarian reserve (16).

Another aspect to consider is the quality of life of women suffering of endometriosis (and adenomyosis): these patients often have lower health-related quality of life (HrQoL) compared to women without endometriosis (17, 18). This psychological aspect has to be connected also to the need of IVF technique due the lower ovarian reserve, showed by low value of AMH (19–22). A counseling to evaluate these aspects before starting an IVF technique is mandatory.

Two recent meta-analysis on adenomyosis (23, 24) evaluated the association of adenomyosis with fertility, pregnancy and neonatal outcomes: adenomyosis is associated with negative effects on fertility after IVF and (independently of the mode of conception) with adverse pregnancy and neonatal outcomes. A proper counselling before IVF and close monitoring of pregnancy in patients with adenomyosis should be recommended.

Some recent works (25, 26) also underlined the underestimated risk of endometrial cancer in patients affected by adenomyosis: they conclude that a close follow-up is mandatory in women affected by endometriosis and, particularly, adenomyosis.

Our study tried to summarize the latest evidences on the correlation between AMH and adenomyosis: the number of scientific works published in the last five years are low, but the results are promising. Our mini-review showed that surgical treatment of adenomyosis (adenomyomectomy, HIFU) does not influence AMH levels and thus has a negligible role on ovarian function.

The strength of this work is the originality of the argument in the field of endometriosis; the main limitation is the heterogeneity of the results in the scientific works, and the relative low number of retrieved publications. Further research on this topic is needed.

## Conclusion

The available evidence shows an unclear relationship between adenomyosis and AMH. Probably, women with adenomyosis have lower levels of AMH and the surgical treatment (adenomyomectomy, HIFU) does not alter this characteristic, so in all likelihood ovarian function is not influenced. Furthermore, a molecular pathology study found that in adenomyotic tissue there is a higher expression of AMHRII, thus we can speculate a possible use, in the future, of AMH as a therapy for adenomyosis.

## Author contributions

All authors contributed to the article and approved the submitted version.

## Conflict of interest

The authors declare that the research was conducted in the absence of any commercial or financial relationships that could be construed as a potential conflict of interest.

## Publisher’s note

All claims expressed in this article are solely those of the authors and do not necessarily represent those of their affiliated organizations, or those of the publisher, the editors and the reviewers. Any product that may be evaluated in this article, or claim that may be made by its manufacturer, is not guaranteed or endorsed by the publisher.
